# Supplementary material for: Methodologies and MR Parameters in Quantitative Magnetic Resonance Neurography: A Scoping Review Protocol
Source: Methods Protoc. 2022 May 6;5(3):39. doi: 10.3390/mps5030039 (PMC9149994; doi:10.3390/mps5030039)
Supplement: Supplementary file 1 [file mps-05-00039-s001.zip › S2_search-query.pdf]

The search queries for all four databases are available online at https://github.com/fabianbalsiger/qmrn-review. As an example, the search query for MEDLINE (PubMed) is presented below, which is equal to commit 55cb5fa in the repository.

```
(
(
"Peripheral Nerves/diagnostic imaging"[majr] OR
"Peripheral Nervous System/diagnostic imaging"[majr] OR
"Peripheral Nervous System Diseases/diagnostic imaging"[majr] OR
"peripheral nerve*"[tiab] OR
"Peripheral Nervous System"[tiab] OR
"Peripheral Nervous System Disease*"[tiab] OR
"peripheral neuropath*"[tiab]
) AND
(
"magnetic resonance imaging"[majr] OR
"magnetic resonance imaging"[tiab] OR "MRI"[tiab] OR
"nuclear magnetic resonance"[tiab] OR "NMR"[tiab] OR
"magnetic resonance neurography"[tiab] OR "MRN"[tiab] OR
"Diffusion Tensor Imaging"[majr] OR
"diffusion tensor imaging"[tiab] OR "DTI"[tiab]
) AND
(
"t1 relaxation time"[tiab] OR
"t2 relaxation time"[tiab] OR
"relaxation time"[tiab] OR
"proton density"[tiab] OR
"magnetization transfer"[tiab] OR
"magnetisation transfer"[tiab] OR
"diffusion tensor imaging"[tiab] OR
"DTI"[tiab] OR
"dixon"[tiab] OR
"perfusion"[tiab] OR
```

```
"susceptibility"[tiab] OR
"segmentation"[tiab] OR
"cross-sectional area"[tiab] OR
"morphometry"[tiab] OR
"radiomics"[tiab] OR
"microstructure"[tiab]
)
) AND
(english[Filter])
```
